# SURF: Direction-Optimizing Breadth-First Search Using Workload State on GPUs

**DOI:** 10.3390/s22134899

**Published:** 2022-06-29

**Authors:** Daegun Yoon, Sangyoon Oh

**Affiliations:** Department of Artificial Intelligence, Ajou University, Suwon 16499, Korea; kljp@ajou.ac.kr

**Keywords:** direction-optimizing BFS, frontier workload, GPU

## Abstract

Graph data structures have been used in a wide range of applications including scientific and social network applications. Engineers and scientists analyze graph data to discover knowledge and insights by using various graph algorithms. A breadth-first search (BFS) is one of the fundamental building blocks of complex graph algorithms and its implementation is included in graph libraries for large-scale graph processing. In this paper, we propose a novel direction selection method, SURF (Selecting directions Upon Recent workload of Frontiers) to enhance the performance of BFS on GPU. A direction optimization that selects the proper traversal direction of a BFS execution between the push and pull phases is crucial to the performance as well as for efficient handling of the varying workloads of the frontiers. However, existing works select the direction using condition statements based on predefined thresholds without considering the changing workload state. To solve this drawback, we define several metrics that describe the state of the workload and analyze their impact on the BFS performance. To show that SURF selects the appropriate direction, we implement the direction selection method with a deep neural network model that adopts those metrics as the input features. Experimental results indicate that SURF achieves a higher direction prediction accuracy and reduced execution time in comparison with existing state-of-the-art methods that support a direction-optimizing BFS. SURF yields up to a 5.62× and 3.15× speedup over the state-of-the-art graph processing frameworks Gunrock and Enterprise, respectively.

## 1. Introduction

**Figure 1:**
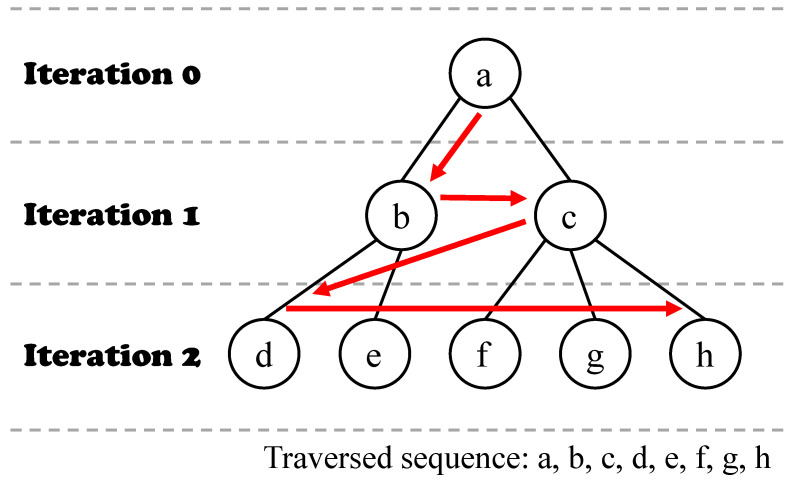
Example of breadth-first search. Red arrows indicate the traversal sequence during BFS.

Graph data structures have been used in a wide range of scientific applications, and engineers and scientists analyze graph data to discover knowledge and insights by using various graph algorithms. Thus, it is important to select appropriate graph algorithms for graph analytic processes and facilitate the process. In many complex graph algorithms, breadth-first search (BFS) is a key building block of them. The BFS is an iterative algorithm, which is initiated on a source vertex and visits all its neighbors. In the following iteration, all vertices visited at the previous iteration become new sources of that iteration, and all neighbors of them are visited. The algorithm is terminated when all reachable vertices are visited. Figure 1 presents how BFS works. This iterative procedure consists of the basic operations such as inspecting neighbors of a vertex and expanding a set of frontiers (i.e., vertices visited at the previous iteration) of each iteration. These two operations are also applied to algorithms such as label propagation, which can be used to detect fraud in commercial transactions [1], and PageRank, which can be used to measure the objective reputation of a certain website [2]. Thus, it is crucial to understand the BFS execution mechanism and characteristics to enhance the performance of BFS itself as well as expand its use to other graph algorithms.

The performance of a BFS in GPU environments can be enhanced further by exploiting parallelism [3,4,5,6,7,8,9]. For example, the workload used to inspect the neighbors is proportional to the number of edges to be checked at each iteration, and the operation can be parallelized. Thus, using thousands of GPU cores for parallelism helps the graph traversal inspect the neighbors with a reduced execution time, achieving a high level of performance.

Even if the parallelism of the GPU helps the overall performance of a BFS, such performance could still be degraded by the huge workload at some parts of the entire traversal. The workload of a BFS is determined by the number of frontiers and can therefore fluctuate. In their well-known publication [10], Beamer et al. observed that the conventional BFS algorithm used to inspect the neighbors of the frontiers is ineffective when the number of frontiers is large. To address this problem, the authors proposed a direction-optimizing BFS that adopts two variations (i.e., directions) of a BFS algorithm: a push (conventional BFS) and a pull. In the pull phase, the active vertices are those not visited until the current iteration is reached, unlike in the push phase, and they are checked to determine whether their neighbors are frontiers. Consequently, an inspection can succeed with a high probability if the number of frontiers is large. Because it showed a performance enhancement in terms of the BFS execution, the direction-optimizing scheme has become popular with BFS schemes.

However, the determination of the direction of a BFS at each iteration or criteria of the selecting directions has not been discussed thoroughly. Many state-of-the-art graph processing frameworks [4,8,10] include a direction-optimizing BFS. However, their methods for determining the direction are naive and have drawbacks in their direction selecting decisions. Because these methods do not handle the varying workload efficiently, they make inappropriate directional decisions at the dataset level (i.e., different datasets) and phase level (i.e., within a single BFS execution). Consequently, the performance can easily degrade. Moreover, the methods yield a high computational overhead, and the overall execution time of the BFS is increased.

In this study, we introduce four workload state metrics and analyze the effect of each metric on the performance of a BFS. Based on our study on new metrics, we propose a novel direction-optimizing method, called Selecting the direction Upon Recent workload of Frontiers (SURF), that handles the workload of each iteration of execution with consideration of new metrics that represent the workload states of the frontiers. The proposed SURF utilizes the metrics as features of the MLP model to predict the label of the direction. The contributions of our study are as follows:

- We propose new workload state metrics and analyze their impact based on theoretical proofs.
- We propose a novel direction-optimizing scheme, i.e., SURF, based on the observation of the new metrics and provide the source code of our proposed scheme, which is publicly available at https://github.com/kljp/SURF/ (accessed on 18 March 2022).
- To measure the effectiveness of the proposed SURF, we provide thorough experiment results using public datasets collected from various perspectives.

The remainder of this paper is organized as follows. Section 2 introduces the background of a direction-optimizing BFS and previous studies related to this topic. Section 3 introduces the workload state metrics and provides theoretical proof to observe their impact on the performance of BFS. Section 4 presents the details of the proposed SURF implementation. Section 5 presents the experiment results, focusing mainly on comparisons between SURF and existing approaches. Finally, we provide some concluding remarks in Section 6.

## 2. Preliminaries

In this section, we briefly introduce the concept of a direction-optimizing BFS and present graph processing frameworks that provide such a method.

### 2.1. Direction-Optimizing BFS

**Figure 2:**
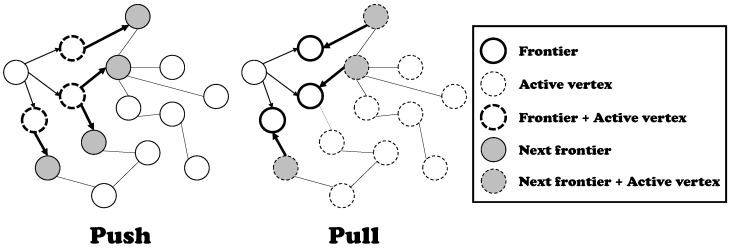
Push and pull mechanism in direction-optimizing BFS.

Based on an observation by Beamer et al. [10], the performance of a BFS algorithm depends on the number of frontiers. The authors first introduced the concept of a direction-optimizing BFS, and Algorithm 1 presents its pseudocode. Their technique supports two variations of the graph traversal method, push and pull phases, as presented in Figure 2. A push is the conventional algorithm of a BFS, that is, its purpose is to find the children of each active vertex. In the push phase, the active vertices are frontiers, and all neighbors of each active vertex are checked to determine whether they have been visited. The neighbors revealed as unvisited become the frontiers (i.e., active vertices) of the next iteration. Algorithm 2 presents the pseudocode of the push phase.

However, in the pull phase, the active vertices are those not visited until the current iteration. The purpose of the pull phase is to find a parent of each active vertex, that is, if any neighbor of a vertex is revealed as a parent, the remaining neighbors of that vertex are not going to be checked because only one parent exists for any vertex. Thus, the noteworthy difference between push and pull is the role of frontiers. In the push phase, frontiers are treated as source vertices of each iteration. However, in the pull phase, unvisited vertices act as source vertices of each iteration, and their neighbors are inspected for whether they are frontiers. Algorithm 3 presents the pseudocode of the pull phase. Note that the performance can be enhanced largely by neighbor inspection skipping, which is presented in line 8.

**Algorithm 1** Direction-optimizing BFS.

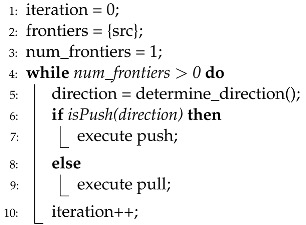



**Algorithm 2** Push-style BFS.

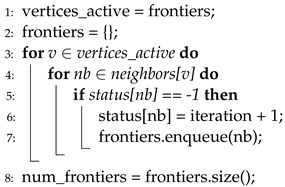



**Algorithm 3** Pull-style BFS.

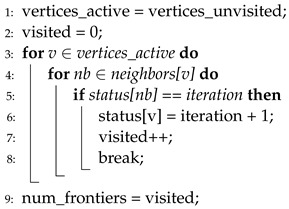



### 2.2. Related Studies

In this study, we present our analysis of three graph processing frameworks that support a direction-optimizing BFS, i.e., hybrid-BFS (HBFS) [10], Gunrock [4], and Enterprise [8]. We describe how each framework determines the direction of the BFS execution, which corresponds to line 5 of Algorithm 1, and our analysis of the drawbacks of each framework in terms of the prediction accuracy and computational overhead required to predict the direction.

HBFS [10] is the first approach that adopts the concept of directional changes in a BFS execution, and Algorithm 4 presents the pseudocode of the direction-optimizing method in HBFS. In HBFS, the direction is changed from a push to a pull when the number of edges of the frontiers is large. In HBFS, the degree of “how large” is determined by a predefined threshold. By contrast, when the number of frontiers is small, the direction changes from a pull to a push. HBFS uses thresholds (i.e., α and β at lines 4 and 5) that are overtuned to several graph datasets. Moreover, high computational overhead is required to calculate the number of edges of the frontiers (line 9).
**Algorithm 4** Direction-optimizing BFS in HBFS.
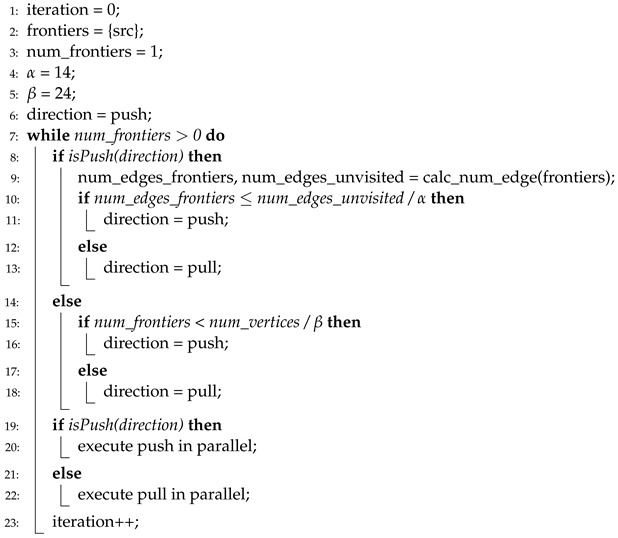


Algorithm 5 presents the pseudocode of the direction-optimizing method in Gunrock [4]. In Gunrock, the conditions for selecting direction are similar to that of HBFS, that is, predefined thresholds (i.e., α and β at lines 4 and 5) are used to select directions. However, Gunrock uses only the number of frontiers, not the number of edges of frontiers (lines 8 and 9). Thus, there is no extra overhead added because the number of frontiers is the basic metric in a BFS, regardless of whether direction-optimization is used. Instead, Gunrock has the same issue in terms of the accuracy of the direction prediction owing to the overtuned thresholds.

Algorithm 6 presents the pseudocode of the direction-optimizing method in Enterprise [8]. Enterprise uses the number of hub vertices (i.e., vertices that have many more neighbors than the average) to select direction. If the number of hub vertices among the frontiers is larger than 30% of all hub vertices, the direction is determined as a pull, whereas if it is smaller than 30%, it is determined as a push. However, Enterprise also shows an inconsistent level of accuracy when tested on various graph datasets owing to a predefined threshold value (30%), which is presented in line 4. Moreover, high overhead is required to calculate the number of hub vertices. This overhead is divided into two terms: (1) Enterprise should calculate the number of hub vertices in a graph before the first iteration (line 5), and (2) the number of hub vertices among frontiers at every iteration (line 7). Thus, the total computational overhead is significant.

In this study, we propose a novel direction-optimizing method to overcome the shortcomings of existing methods in terms of the prediction accuracy and computational overhead of the direction prediction.
**Algorithm 5** Direction-optimizing BFS in Gunrock.
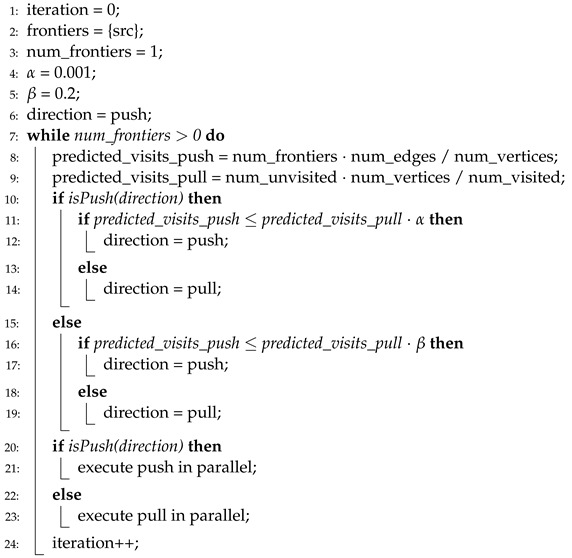


**Algorithm 6** Direction-optimizing BFS in Enterprise.

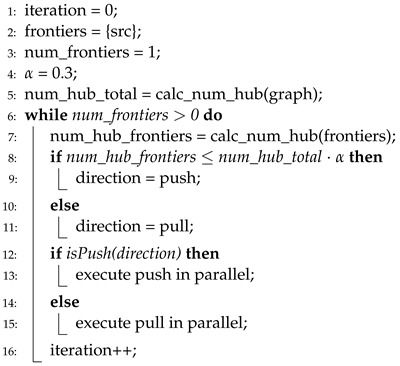



## 3. Can “Workload State” Help Achieve Efficient Direction Selections in BFS?

The workload changes at each iteration of the BFS algorithm execution, and we hypothesize that such changes can be used to achieve efficient direction selections in a BFS. In this section, we define four metrics that describe workload state and give the physical meaning of them. Using these metrics, we describe our hypotheses and provide a logical proof.

The changes defined as the “workload state” in this study are used to represent the fluctuation level of the number of frontiers ($n_{f}$). The workload state is significantly affected by the increase in $n_{f}$, and the sharpness of $n_{f}$ changes. Because the performance of a push and pull phase depends largely on the change in $n_{f}$ in the direction-optimizing BFS, it is important to understand the behavior of the changes in the workload state for selecting the traversal direction. In this study, we introduce four metrics derived from $n_{f}$ to illustrate the relationship between the workload state and the traversal direction.

Let $n_{f}^{x}$ be the number of frontiers at iteration *x*. We then define $s_{f}$ as the variation of $n_{f}$; in particular, $s_{f}^{x}$ denotes the variation of $n_{f}$ from iteration x−1 to *x* (x≥0), as follows:

(1) $$s_{f}^{x}=\left\{\begin{array}{cc} \Delta n_{f}^{x}=n_{f}^{x}-n_{f}^{x-1} & \mathrm{if}\;x>0\\ n_{f}^{x} & \mathrm{else} \end{array}\right..$$

**Figure 3:**
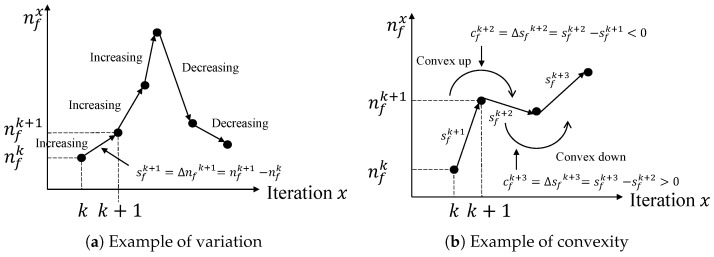
Metrics with respect to the number of frontiers used to determine workload state.

We determine whether $n_{f}$ increases by observing the sign of $s_{f}$ (i.e., a positive value indicates an increase, and a negative value indicates a decrease). Figure 3a shows a plot after iteration *k*. A positive $s_{f}^{k+1}$ implies that $n_{f}$ has increased, whereas a negative $s_{f}^{k+4}$ implies that $n_{f}$ has decreased. Based on this observation, we found that the metric $s_{f}$ can be related to the performance of the push phase (i.e., using $s_{f}$ in the push phase to achieve a higher performance).

**Observation** **1.** *As $s_{f}$ increases, the cost of the push phase also increases.*

Let $n_{t}$ be the maximum number of threads assigned to the physical cores of the GPU. It is assumed that each thread takes a frontier from the frontier set. In the CUDA [11] architecture, 32 threads are executed as a warp unit. To execute the next instruction, all 32 threads must complete the current instruction. Thus, each warp must complete work on the current 32 frontiers before taking the next 32 frontiers (synchronization barrier). Based on this principle, we define $C_{sync}^{x}=n_{f}^{x}/n_{t}$ as the cost of the synchronization barrier at iteration *x* because each warp requires $C_{sync}^{x}$ times of synchronization barrier to finish the job at iteration *x* [9]. We then define the increased cost from iteration x−1 to *x* as follows:

(2) $$\begin{array}{cc} C_{incr}^{x} & =C_{sync}^{x}-C_{sync}^{x-1}\\ \; & =\frac{n_{f}^{x}-n_{f}^{x-1}}{n_{t}}\\ \; & =\frac{s_{f}^{x}}{n_{t}}. \end{array}$$

Cost $C_{incr}^{x}$ increases as $s_{f}^{x}$ increases. Therefore, the cost of the push phase increases as $s_{f}$ increases.

Nevertheless, $s_{f}$ only helps to determine whether $n_{f}$ increases. To understand in detail how the workload changes, we should determine how sharply $n_{f}$ changes or whether the sign of $s_{f}$ has changed. Thus, we also define $c_{f}$ as the variation of $s_{f}$, where $c_{f}^{x}$ denotes the variation of $s_{f}$ from iteration x−1 to *x* (x≥0), as follows:

(3) $$c_{f}^{x}=\left\{\begin{array}{cc} \Delta s_{f}^{x}=s_{f}^{x}-s_{f}^{x-1} & \mathrm{if}\;x>0\\ s_{f}^{x} & \mathrm{else} \end{array}\right..$$

Metric $c_{f}$ represents the convexity of the graph for $n_{f}$ on a Cartesian plane. Similar to $s_{f}$, we determine the direction in which the graph for $n_{f}$ is convex by observing the sign of $c_{f}$ (i.e., positive is convex downward and negative is convex upward). Figure 3b shows a plot of the traversals in which the convexity of the graph changes in a row after iteration *k*. The graph shows that $s_{f}$ decreases after iteration k+1. Thus, the shape of the graph appears to be convex upward. However, $s_{f}$ increases after iteration k+2, and thus the graph has a convex downward shape. Based on this observation, we found that the metric $c_{f}$ can be related to the performance of the push phase, such as $s_{f}$.

**Observation** **2.** *As $c_{f}$ increases, the cost of the push phase also increases.*

From the definition of $C_{incr}^{x}$, we deduce another equation as follows:

(4) $$\begin{array}{cc} C_{incr}^{x} & =\frac{s_{f}^{x}}{n_{t}}\\ \; & =\frac{c_{f}^{x}+s_{f}^{x-1}}{n_{t}}. \end{array}$$

In this equation, $s_{f}^{x-1}$ is constant because the value of $s_{f}^{x-1}$ has already been determined at iteration x−1. Thus, cost $C_{incr}^{x}$ increases as $c_{f}^{x}$ increases, i.e., the cost of the push phase increases as $c_{f}$ increases.

To determine the ratio of the current frontiers of the entire traversal, we define $r_{f}=n_{f}/n_{v}$ as the workload occupancy at each iteration, where $n_{v}$ is the number of vertices in a graph. The metric $r_{f}$ is related to the performance of the pull phase.

**Observation** **3.** *As $r_{f}$ increases, the performance of the pull phase also increases.*

We define the probability that the inspected neighbor is the frontier as follows:

(5) $$p_{f}=\frac{n_{f}}{n_{v}-1}.$$

Let $d_{u}^{k}$ be the degree of the *k*th vertex among unvisited vertices. We then define the probability that the parent of that vertex is discovered at the current iteration as follows:

(6) $$\begin{array}{cc} p_{f}^{k} & =1-{p_{f}}^{0}\cdot {\left(1-p_{f}\right)}^{d_{u}^{k}}\\ \; & =1-{\left(1-p_{f}\right)}^{d_{u}^{k}}. \end{array}$$

We then define the probability that the parents of all unvisited vertices are discovered at the current iteration, where $n_{u}^{x}$ is the number of unvisited vertices at iteration *x* (the current iteration), as follows:

(7) $$p_{f}^{u}=\prod_{j=0}^{n_{u}^{x}-1}\left(1-{\left(1-p_{f}\right)}^{d_{u}^{j}}\right).$$

Thus, $p_{f}^{u}$ increases with $p_{f}$. Because the pull phase is effective when as many parents as possible are discovered, based on the definition of $p_{f}^{u}$, the performance of the pull phase increases as $p_{f}^{u}$ increases. Furthermore, $p_{f}$ is proportional to $r_{f}$. Therefore, the pull phase becomes more effective when $r_{f}$ increases.

In addition, we define $n_{u}$ as the number of unvisited vertices, and $n_{u}^{x}$ denotes the value of $n_{x}$ at iteration *x*; thus, $n_{u}^{x}$ is defined as follows:

(8) $$n_{u}^{x}=n_{v}-\sum_{i=0}^{x}n_{f}^{x}.$$

We also define $r_{u}=n_{u}/n_{v}$ to indicate how many vertices remain to be explored at the following iterations as ratio, particularly $r_{u}^{x}=n_{u}^{x}/n_{v}$ at iteration *x*. Thus, we can inspect where the current iteration is located using $r_{u}$, for example, whether the current iteration is located in the early, middle, or late stages of the entire traversal. Based on this observation, we found that metric $r_{u}$ is related to the performance of the pull phase.

**Observation** **4.** *As $r_{u}$ decreases, the performance of the pull phase increases.*

Let *t* be the time required to inspect one neighbor. Using *t*, we define $T^{k}$, the expected time to inspect the neighbors to find a parent of the *k*th vertex among unvisited vertices at the current iteration as follows:

(9) $$T^{k}=\sum_{i=0}^{d_{u}^{k}-1}\left(p_{f}\cdot {\left(1-p_{f}\right)}^{i}\cdot \left(i+1\right)\cdot t\right).$$

Thus, the higher $d_{u}^{k}$ is, the higher $T^{k}$ becomes. It is assumed that each thread takes a vertex from the unvisited vertices. We then define $n_{cycle}=\lceil n_{u}^{x}/n_{t}\rceil$ as the number of vertices that each vertex must process. Using $n_{cycle}$, we also define *T*, the time expected to process all unvisited vertices at iteration *x*, where $J_{h}$ is the integer interval $\left[h\cdot n_{t},h\cdot n_{t}+\left(n_{t}-1\right)\right]$, as follows:

(10) $$T=\sum_{h=0}^{n_{cycle}-1}\max_{j\in J_{h}}T^{j}.$$

Regardless of each fraction of the sum, the smaller $n_{cycle}$ is, the smaller *T* becomes. Therefore, the running time of the pull phase decreases as $r_{u}$ decreases ($n_{cycle}=\lceil r_{u}^{x}\cdot n_{v}/n_{t}\rceil$).

In summary, we classified the four workload state metrics into two categories based on their relationship with the traversal direction. First, the fluctuations in $s_{f}$ and $c_{f}$ are directly related to the performance of the push phase, as presented in Observations 1 and 2. They are used to describe the variation in $n_{f}$ during the entire traversal process and are sensitively affected by the degree of change in the workload, such as explosive increases or drastic decreases in $n_{f}$. Thus, it is important to determine whether the push phase is sufficiently effective at handling the current workload using $s_{f}$ and $c_{f}$. Second, $r_{f}$ and $r_{u}$ can be applied as important factors for measuring the performance of the pull phase. The metric $r_{f}$ may be crucial for determining the effectiveness of the pull phase in terms of the success of the parent inspection, as presented in Observation 3. By contrast, metric $r_{u}$ is crucial for determining the effectiveness in terms of the length of the running time of the pull phase, as presented in Observation 4. We can therefore conclude that $s_{f}$ and $c_{f}$ are crucial parameters for measuring the expected performance during the pull phase.

By observing the workload state with $s_{f}$, $c_{f}$, $r_{f}$, and $r_{u}$, the BFS algorithm can determine which traversal direction is more effective for the performance at the current iteration. Our proposed direction-optimizing method, SURF, adopts these workload state metrics as features for predicting the BFS direction. In the next section, we describe the details of SURF implementation.

## 4. SURF Implementation

**Figure 4:**
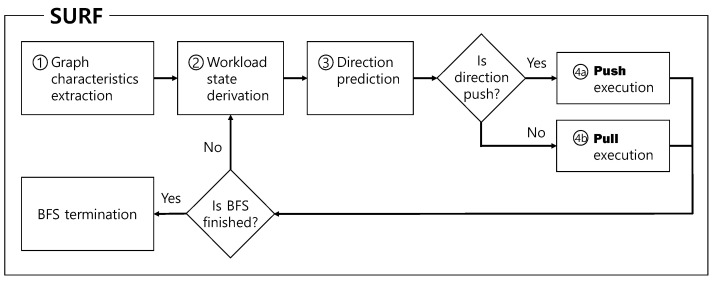
Workflow of SURF.

Based on our findings, we built our BFS execution implementation, SURF, and Figure 4 shows the workflow of SURF. In our SURF, the search direction (i.e., whether pull or push) is selected based on the input features, including the workload state. We describe the details of other input features in Section 4.2.

### 4.1. Use of MLP for Applying Workload State to Select a Direction

There are three possible options for implementing our findings described in Section 3. First, designing a rule-based direction-prediction algorithm is the simplest option. With this option, the direction is selected based on multiple conditions that test the input feature values. This method predicts the direction with low computational overhead. However, the accuracy is not guaranteed because the hyperparameters of conditions can be overtuned to several existing graphs, that is, they are prone to overfitting. Second, a statistical method is available for predicting the label of a direction. We can estimate how close the value of each feature at the current iteration is to that of the collected data. We then select the label that shows smaller feature errors as the direction. This method yields a certain level of accuracy. However, it cannot be used to analyze the collected data at every iteration during runtime (i.e., an online analysis) because it is too heavy to use statistical methods even if sampling is applied.

However, various machine learning methods (i.e., the third option) are available to predict the label of the direction in terms of both the accuracy and computation time. In contrast to rule-based methods, machine learning techniques are more robust to hyperparameter overtuning for several graphs. Moreover, we can achieve high accuracy with incomparably smaller computation time for predicting the labels than statistical methods when we have a trained model.

There are also a few candidates for machine-learning methods, including decision trees, naive Bayes, logistic regression, and deep neural networks. Although a decision tree is simple, it is prone to overfitting without troublesome branch pruning [12,13]. Naive Bayes is also difficult to use effectively because it requires the features independent of each other [14]; however, features are dependent in this study. In addition, our direction prediction requires the decision boundary more elaborated (e.g., provided by hidden layers) than that of logistic regression [15] to deal with various kinds of graph datasets. Deep neural networks (DNNs) are a popular choice for many recent applications. However, not all are adequate for our purpose. Deep neural networks with multiple hidden layers have a vast number of calculations for a label prediction. Moreover, the larger number of hidden layers does not guarantee a high accuracy because only six features are used in SURF. Instead, a multilayer perceptron (MLP) has fewer layers that can achieve the same level of accuracy as deep neural networks with many hidden layers when the number of features is small [16]. We used an MLP with one hidden layer for the direction prediction of SURF because it requires only a series of simple matrix multiplications to draw a label of the direction.

### 4.2. Feature Description

In Section 3, we show that $s_{f}$, $c_{f}$, $r_{f}$, and $r_{u}$ are the features used to describe the workload state. In addition to these four features, we added two additional features to the current implementation of SURF to predict the BFS direction in SURF, and the descriptions of input features are listed in Table 1. In this subsection, we present the details of these two additional features, $m_{d}$ and $p_{h}$.

To obtain more detailed characteristics of the input graph, we utilized two extra features that vary based on the graph, i.e., $m_{d}$ and $p_{h}$. The $m_{d}$ feature is the average degree of vertices in a graph, that is, $m_{d}=n_{e}/n_{v}$, where $n_{v}$ and $n_{e}$ are the numbers of vertices and edges in the graph, respectively. The $p_{h}$ feature represents the probability of the hub vertex and skewness of the graph. The skewness of the graph can be classified into two shapes according to the value of $p_{h}$. If $p_{h}<0.5$, the distribution of the vertex degrees has a right-skewed shape (e.g., social networks). However, the distribution of the vertex degrees is left-skewed if $p_{h}>0.5$, for example, in road networks. Therefore, it is beneficial to determine the value of $p_{h}$ to identify the shape of the degree distribution of the input graph. SURF derives $p_{h}$ by sampling vertices to reduce the computational time, and the sampling size is set using a formula [17,18] with a 95% confidence level and 5% margin of error. By adding these two extra features, SURF can predict the direction more accurately.

### 4.3. Model Training

To collect training data, we chose 51 graph datasets available at Network Data Repository [19] and SuiteSparse Matrix Collection [20]. Our datasets consist of various types of networks including social networks, web graphs, road networks, technological networks, citation networks, biological networks, and synthetic graphs.

To train the MLP model, we used Keras 2.4.3 [21] and TensorFlow 2.4.1 [22] in Python 3.9.7. For training our MLP model, we built a data module in C++ to collect and label the data automatically. This module executes both directions multiple times at each iteration of BFS and determines the label (*L*) of the current iteration as the direction that reported a better average performance than the other direction. Then, one record is generated, including six features and a label at each iteration, following the format of $\left\{m_{d},p_{h},s_{f},c_{f},r_{f},r_{u},L\right\}$. Thus, the number of records generated through the entire traversal is the same as the total length of the iterations. To provide a vast number of records to the MLP model, we iterated this workflow for each dataset. Therefore, we collected 10,224,848 records for the training model from 51 graph datasets, that is, approximately 200,000 records were generated for each graph.

To validate the accuracy of the label prediction (i.e., whether a push or pull) of our trained model, we chose 14 graph datasets available at the same repositories as the training data [19,20]. These graph datasets were not included in the graph suite for the training data. We did not apply any rigorous rules to split the graph datasets into training and validation datasets. Thus, we simply divided the datasets at a ratio of 4:1.

Our trained MLP model achieved 93% accuracy in label prediction from our validation dataset. To enable a rapid prediction of the direction in BFS runtime, we extracted the exact values of the weights and biases from our trained model using the Keras APIs “load_model” and “get_weights” [23]. The extracted values are used to predict a label of direction as a series of simple matrix multiplications written in C++; thus, the times required for loading the model architecture and extra jobs are excluded. This direction-predicting model is used to determine whether the pull phase should be executed at each iteration of the BFS in SURF.

### 4.4. Detail of Implementation

**Figure 5:**
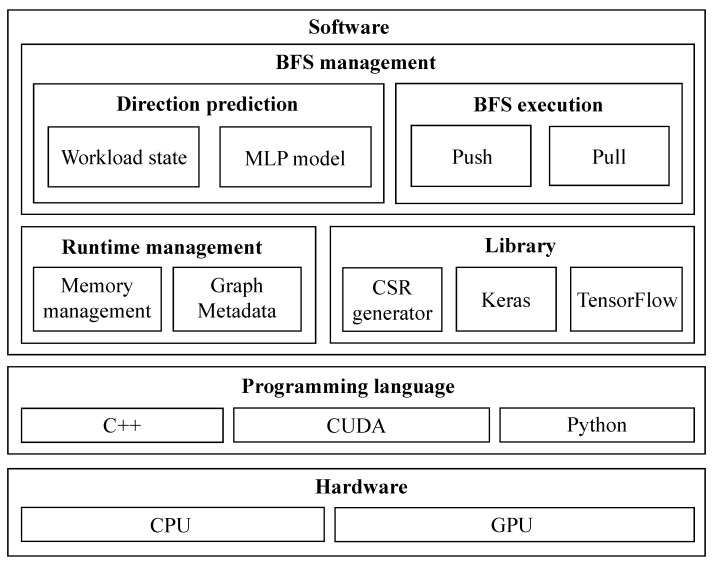
SURF architecture.

Figure 5 presents the overview of SURF implementation. Before initiating SURF, the following prerequisites are required: MLP model training, and graph dataset preprocessing. As mentioned in Section 4.3, the MLP model of SURF is trained with six input features, and one direction (i.e., push or pull) as output, using Keras [21] and TensorFlow [22]. As the input of BFS, one graph dataset is required to be transformed into a compressed sparse row (CSR) representation [24] for memory-efficient graph processing. The trained MLP model and CSR of the graph dataset are used as inputs of SURF.

When SURF is initiated with inputs, memory management allocates memory space of graph data structure using CSR of the graph. Based on the graph data structure, characteristics (i.e., $m_{d}$ and $p_{h}$) of the input graph are extracted. After that, the first iteration of BFS is initiated. At each iteration, four workload state metrics (i.e., $s_{f}$, $c_{f}$, $r_{f}$ and $r_{u}$) are derived with simple calculation (e.g., equations for variation and convexity). In turn, two graph characteristics and four workload state metrics are used as input features of the trained MLP model, and the label of output is determined as the direction of that iteration. Like the direction, the corresponding variation of BFS is executed. This process is repeated until BFS is terminated (i.e., when the number of frontiers is zero). Algorithm 7 presents the pseudocode of this iterative process of SURF. The noteworthy difference between SURF and existing direction-optimizing BFS is presented in line 6 of Algorithm 7. Line 6 presents that SURF derives the latest state of workload at every iteration to deal with changing workload flexibly.
**Algorithm 7** Workload state-based direction-optimizing BFS.
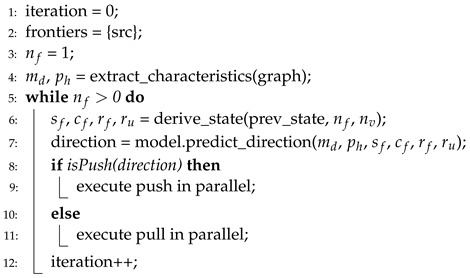


## 5. Evaluation

### 5.1. Experimental Setup

The implementation of SURF is written in C++ and CUDA [11]. The source code was compiled with NVIDIA nvcc compiler (version 11.5) [25] with the -O3 optimization flag. We conducted all experiments in this study on Ubuntu 20.04.2 LTS server equipped with an Intel Core i7-8700 CPU (3.20GHz) and 32GB memory. We used NVIDIA RTX 3080 GPU (8704 CUDA cores and 10GB DRAM capacity) as the accelerator.

In our evaluation, we utilized eight graph datasets that were not applied to train the MLP model. Table 2 lists the details of the graph suite used for the evaluation, all of which are abbreviated for simpler indications.

In Section 5.2 and Section 5.3, we compare the experimental results of the direction-optimizing methods between SURF and other state-of-the-art frameworks. In these experiments, each framework used its own direction-optimizing method. However, all frameworks use the same BFS implementation to make a fair comparison. That is, the differences in the experiment results depend only on their direction-optimizing methods. By contrast, in Section 5.4, we compare the overall performance of the BFS implementation including the direction-optimizing method between frameworks. We measure the performance using their actual implementations from public repositories [26,27]. Each reported numerical value in our experiments is an average of 1024 iterative runs.

### 5.2. Performance of Direction Prediction

To evaluate the effectiveness of the direction prediction of SURF, we defined two criteria in this subsection: the accuracy of the prediction and the reduced execution time. The accuracy represents the ratio of correct predictions during the entire traversal process. During this experiment, the correct direction was determined as that showing a shorter execution time at each iteration. However, the time reduction through the correct decision for the direction prediction can be subtle, although it is correct at one iteration. However, the amount of time added by incorrect decisions can be large at other iterations. Thus, it is inadequate to evaluate the prediction performance using only a prediction accuracy measure. We consider another metric to measure the duration of the reduced execution time. We defined the reduced execution time as the running time saved by the correct decisions at all iterations.

Table 3 lists the prediction accuracy and reduced execution time of each direction-optimizing method of the state-of-the-art frameworks on the graph datasets for evaluation. Excluding the IC measurement, SURF showed the highest prediction accuracy and a reduced execution time in comparison with the other methods. The lowest standard deviations of the prediction accuracy and the reduced execution time in SURF demonstrate the effectiveness of the SURF scheme as well as its applicability to various graph datasets.

### 5.3. Overhead for Direction Prediction

In this subsection, we measure the computational overhead required to determine the direction of each direction-optimizing method. The computational overhead is defined as an aggregate of the computation time required to determine the direction for all iterations. Table 4 lists the runtime of the BFS executions and the computational overhead for each method. As shown by the zero overhead in the table, Gunrock does not require extra metrics to determine the direction, except for the number of frontiers, that is, the prediction accuracy is quite low compared to the other approaches, as shown in Table 3. Enterprise and HBFS yield a high overhead because they require additional metrics (i.e., the number of hub vertices and the number of edges checked from the frontiers, respectively) to make a directional decision. The time required to calculate the number of edges from the frontiers is proportional to the number of current frontiers. However, all vertices must be checked to determine the number of hub vertices. Thus, the computational overhead of Enterprise is incomparably higher than that of the other methods on datasets with a large number of vertices, such as UU. By contrast, SURF shows only a low overhead, regardless of the dataset. This is because the computational overhead per iteration does not change with the dataset. In SURF, the number of calculations required to predict the direction is fixed because the number of computations between neurons does not change. Thus, the entire computational overhead is proportional to the number of iterations, and not the number of vertices. Consequently, the entire runtime of SURF can be reduced significantly with only a low computational overhead.

### 5.4. Overall Performance of SURF

Table 5 shows the performance of the BFS execution on actual implementations obtained from public repositories [26,27] of the frameworks. SURF outperforms the other frameworks on the datasets, except for IC. For IC, Enterprise shows a slightly higher throughput than SURF because the accuracy and reduced execution time of Enterprise are higher. On average, SURF presents speedups of 2.82× (with a highest speedup of 5.62×) and 1.77× (with a highest speedup of 3.15×) over Gunrock and Enterprise, respectively. These speedups are achieved mainly from the advantages of high accuracy and low computational overhead for predicting the direction.

## 6. Conclusions

In this study, we proposed a direction-optimizing method that utilizes the workload state of the frontiers. We observed that the workload state features defined in this study have a significant impact on the traversal direction at each iteration of the BFS. We verified that the higher accuracy of the proposed method for predicting the label of the traversal direction is based on the features of the workload state. Moreover, the proposed method only yields a lower computational overhead than the previous methods in predicting the direction. We expect that the proposed method will also provide a better direction-selecting decision for other graph processing frameworks.

However, we did not discover the correlation between input features and the results from our trained model due to the black box nature of our model. It could be possible to explore the impact of each feature on the results to further improve the interpretability of our model. We will leave this as our future work.

## Figures and Tables

**Table 1:** Description of input features in SURF.

Notation	Description
$s_{f}$	The variation of $n_{f}$
$c_{f}$	The variation of $s_{f}$
$r_{f}$	The ratio of frontiers
$r_{u}$	The ratio of unvisited vertices
$m_{d}$	The average degree of vertices in a graph
$p_{h}$	The probability of hub vertex

**Table 2:** Graph specifications. $n_{iter}$ is the average of the number of iterations to finish BFS.

Graph Name	Abbr.	$n_{v}$	$n_{e}$	$n_{\mathrm{iter}}$	$m_{d}$	$p_{h}$
soc-LiveJournal1	LJ	4,847,571	137,987,546	14	28.47	0.25
soc-orkut	OR	2,997,166	212,698,418	8	70.97	0.31
soc-pokec	PK	1,632,803	44,603,928	10	27.32	0.32
cit-patent	PT	3,774,768	33,037,894	19	8.75	0.37
bio-mouse-gene	MG	42,923	29,007,800	9	643.28	0.34
bio-human-gene1	HG	22,283	24,691,926	7	1108.11	0.32
socfb-uci-uni	UU	58,790,782	184,416,390	17	3.14	0.07
indochina-2004	IC	7,414,866	388,218,622	27	52.36	0.14

**Table 3:** Accuracy of direction prediction and reduced execution time by direction selecting based on the prediction according to the direction-optimizing methods. The standard deviation is abbreviated as Std. dev. A lower Std. dev is better. The best case among the four methods is listed in bold.

Dataset	Accuracy (%)				Reduced Time (%)			
	SURF	Enterprise	HBFS	Gunrock	SURF	Enterprise	HBFS	Gunrock
LJ	**99.38**	93.48	87.52	55.76	**99.97**	98.19	92.51	82.28
OR	**96.94**	90.25	91.59	63.00	**99.77**	97.93	95.80	85.40
PK	**92.96**	91.35	87.30	51.42	**98.36**	96.84	91.98	76.67
PT	**96.01**	93.14	86.28	56.11	**98.99**	98.36	89.59	75.05
MG	**98.01**	42.46	53.64	89.93	**97.88**	83.32	92.10	79.99
HG	**93.37**	43.78	63.91	92.04	**93.29**	78.06	92.87	84.09
UU	**90.90**	90.39	67.29	47.69	**96.20**	93.77	80.48	78.29
IC	92.10	**94.93**	74.95	23.87	96.51	**98.25**	89.68	68.21
Average	**94.96**	79.97	76.56	59.98	**97.62**	93.09	90.63	78.75
Std. dev	**0.030**	0.228	0.138	0.223	**0.022**	0.079	0.045	0.056

**Table 4:** Computational overhead and runtime. The best case among the four methods is shown in bold.

Dataset	Runtime (ms)				Overhead (ms)			
	SURF	Enterprise	HBFS	Gunrock	SURF	Enterprise	HBFS	Gunrock
LJ	**4.81**	8.19	6.87	8.18	0.04	3.02	0.53	**0**
OR	**3.93**	6.40	5.61	9.02	0.02	1.88	0.24	**0**
PK	**1.68**	2.90	2.26	2.45	0.02	1.11	0.27	**0**
PT	**4.49**	7.20	7.46	8.65	0.04	2.57	0.67	**0**
MG	**0.49**	0.82	0.75	0.55	0.01	0.23	0.18	**0**
HG	**0.36**	0.61	0.51	0.37	0.01	0.17	0.12	**0**
UU	**58.76**	106.38	154.44	130.89	0.03	33.01	4.50	**0**
IC	**17.38**	20.70	21.96	31.21	0.03	4.62	1.37	**0**

**Table 5:** BFS performance measured in GTEPS (billions of traversed edges per second), i.e., the higher the GTEPS, the better the performance. The best performance is indicated in bold.

Framework	LJ	OR	PK	PT	MG	HG	UU	IC
SURF	**29.63**	**56.57**	**27.02**	**7.41**	**60.15**	**69.39**	**3.73**	22.52
Enterprise	20.25	49.27	20.15	5.10	26.38	29.55	1.18	**23.51**
Gunrock	22.59	22.10	26.44	3.50	13.16	15.85	3.65	4.01

## Data Availability

The data presented in this study are publicly available at https://github.com/kljp/SURF/ (accessed on 18 March 2022).
